# A Rare Endobronchial Tumor in a Pediatric Patient

**DOI:** 10.3390/diagnostics14202254

**Published:** 2024-10-10

**Authors:** Pooja Parekh, Ajay Wagh

**Affiliations:** 1 Department of Medicine, University of Chicago, Chicago, IL 60637, USA; pooja.parekh@uchicagomedicine.org; 2 Division of Pulmonary and Critical Care, Department of Medicine, University of Chicago, Chicago, IL 60637, USA

**Keywords:** pediatric endobronchial tumor, central airway obstruction, bronchoscopy

## Abstract

A pediatric patient who presented with non-specific respiratory symptoms, including mild hemoptysis, wheezing, and eventual respiratory distress, was found to have a rare endobronchial inflammatory myofibroblastic tumor obstructing her right mainstem bronchus. It was diagnosed and initially debulked using bronchoscopy, which helped to stabilize the patient and eliminate the need for supplemental oxygen. The patient subsequently underwent successful removal of the residual tumor with parenchymal-sparing sleeve resection. This case highlights the importance of pursuing appropriate imaging along with diagnostic and therapeutic bronchoscopy for an endobronchial lesion to help manage pediatric patients with persistent respiratory symptoms.

**Figure 1:**
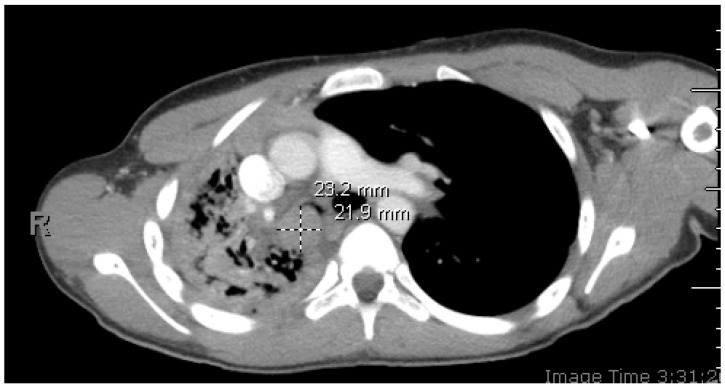
Initial chest CT with contrast: axial image depicting right mainstem bronchus intraluminal mass. A 14-year-old healthy patient presented with cough, congestion, abdominal pain, and chest tightness worsening over several weeks. She denied any contact with people with similar symptoms or recent travels and received all recommended vaccinations. She reported two episodes of hemoptysis about 1.5 years prior. The first episode was associated with cough and wheezing for one day. The second episode was associated with similar symptoms, and she was given antibiotics to treat for infection at that time. Upon examination, the patient was tachycardic with a heart rate of 130 beats/min, tachypneic with a respiratory rate of 30 breaths/min, and hypoxic with an oxygen saturation of 74% on room air, therefore requiring a non-rebreather mask for oxygenation. Lung examination revealed coarse, significantly diminished breath sounds throughout the right side. CT imaging revealed a 2.3 × 1.9 × 3.0 cm soft tissue lesion obstructing the right mainstem bronchus with resultant atelectasis of the right lower lobe, right-sided tracheal deviation with mediastinal shift, and patchy airspace disease throughout the right middle and upper lobes concerning for post-obstructive pneumonia.

**Figure 2:**
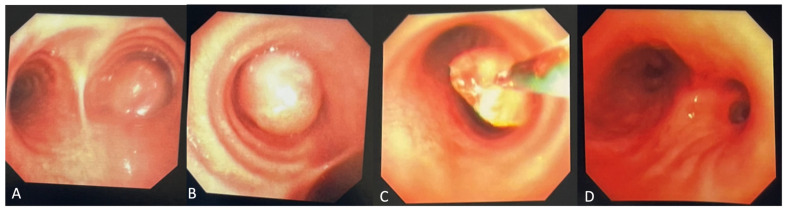
(**A**,**B**) Initial diagnostic bronchoscopy was notable for a smooth, exophytic, partially mobile intraluminal mass with a long base causing central airway obstruction in the right mainstem bronchus. (**C**) The mass was bronchoscopically biopsied and subsequently debulked using electrosurgical snare, forceps, and cryodebulking as well as cryotherapy treatment at the base of the tumor. (**D**) Post-bronchoscopy, the right mainstem bronchus and bronchus intermedius were patent. Endobronchial tumors are extremely rare in the pediatric population, accounting for only up to 1% of all pediatric cancers [1]. In case of involvement of the upper respiratory tract, obstructive symptoms such as stridor, wheezing, and dyspnea appear. Additionally, chronic cough from mucosal irritation and hemoptysis can be observed [2]. With more severe obstruction, bronchiectasis and pulmonary atelectasis could occur. The lack of pathognomonic symptoms can lead to frequent misdiagnoses with other conditions (such as bronchitis, pneumonia, or asthma), potentially contributing to diagnostic delay. Imaging and biopsy remain crucial in the diagnostic workup, with bronchoscopy serving as an effective diagnostic and therapeutic modality.

**Figure 3:**
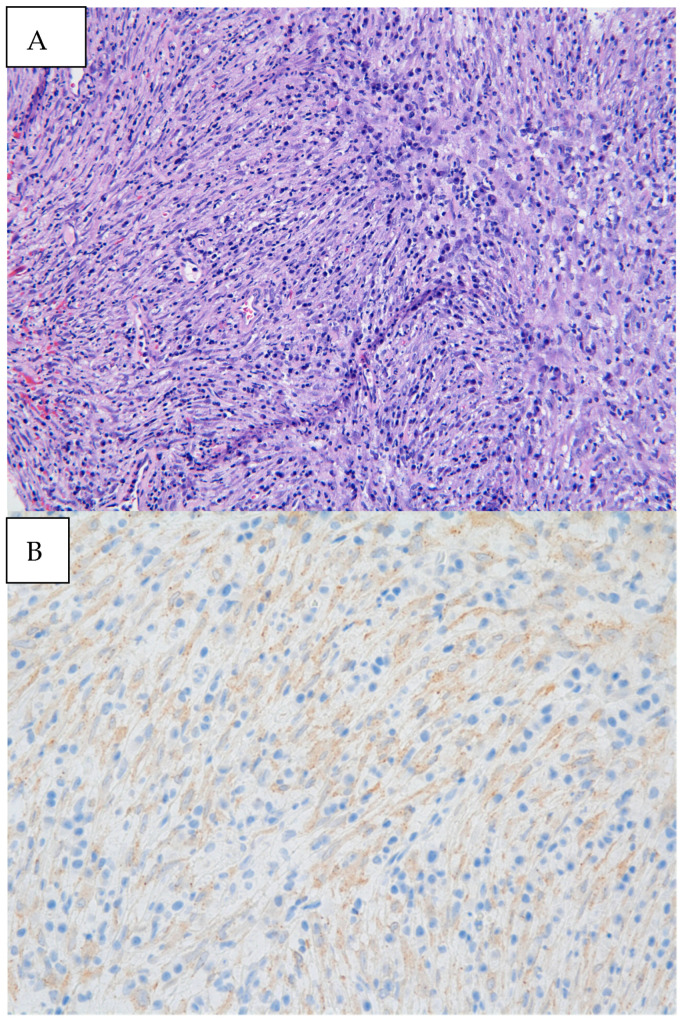
(**A**) Biopsy specimen (20× magnification) retrieved from diagnostic bronchoscopy revealed myofibroblastic spindle cells admixed with plasma cells and lymphocytes. (**B**) Immunohistochemical staining was diffusely weakly positive for ALK, as seen by the granular cytoplasmic staining of spindle cells. Pathology revealed an inflammatory myofibroblastic tumor (IMT) with positive immunohistochemical staining for ALK. Next-generation sequencing was performed on the biopsy material notable for EML4:ALK fusion. IMTs often appear as well-defined, heterogeneous masses with areas of necrosis or hemorrhage. Contrast enhancement can be heterogeneous, reflecting the variable vascularity within the tumor [3]. In a review of pediatric cases of IMTs, subacute dyspnea and hemoptysis were the most common clinical presentations, which may be attributed to tumor growth, irritation of the airway, and bleeding from the vascularity of the tumor itself [4]. Endobronchial involvement may cause partial or complete obstruction of the airway, leading to findings of atelectasis and post-obstructive pneumonia on imaging. Defining pathologic findings of IMT include spindle cell proliferation often in fascicles or whorls in an inflammatory background of plasma cells, lymphocytes, and/or eosinophils [3]. Anaplastic Lymphoma Kinase (ALK) gene rearrangements are more commonly seen in IMTs in the pediatric population, with a particular predilection for pulmonary involvement. ALK receptors and fusion with clathrin heavy chains is seen in up to half of IMTs, providing an opportunity for targeted medical therapy [5]. The clinical course of IMT is variable. It is formally classified as a neoplasm of intermediate malignancy with overall favorable survival, with individuals in up to 5% of cases having distant metastatic disease. Recurrent and metastatic lesions are more commonly seen in abdominopelvic IMTs but have been reported in pulmonary IMTs [5]. Some reports suggest that absent ALK expression is associated with distant metastases and subtle histologic changes such as greater nuclear atypia and atypical mitoses that may reflect poor prognosis [6]. Surgery remains the treatment of choice for localized tumors. Though no guideline has formalized the standard of care for recurrent or metastatic lesions, targeted therapy with ALK inhibitors such as crizotinib, ceritinib, and alectinib has promising impacts on response rates and progression-free survival in ongoing clinical trials [7]. Endobronchial tumor debulking has been widely described as a method for palliating respiratory symptoms due to malignant airway obstruction [8]. Our patient was able to be discharged home one day after initial debulking due to rapid resolution of dyspnea and hypoxia. She underwent repeat bronchoscopy one month later. Residual tumor was seen at the anterior wall of the bronchus intermedius. After a multi-disciplinary case review with our interventional pulmonary, pediatric oncology, and thoracic surgery colleagues, the patient underwent successful parenchymal-sparing surgical resection of the bronchus intermedius which allowed for complete tumor removal without sacrificing pulmonary parenchyma. Surveillance bronchoscopy two months and one year after resection was performed in conjunction with CT imaging every six months, which were without recurrence of the tumor. Given the rarity of pediatric endobronchial tumors, it has been difficult to study and develop a consensus guideline regarding identification, treatment, and surveillance of such tumors. In this case, while imaging provided insight into the location and certain characteristics of the mass, biopsy and debulking remained crucial to both diagnosis and clinical stabilization. Our patient benefitted from an initial diagnostic and therapeutic bronchoscopy which led to the formal diagnosis, an improvement in hypoxia in the critical care setting, adequate time for surgical planning, and surveillance after resection. Bronchoscopic evaluation and management allows for a patient stabilization and provides guidance in further management strategies.

## Data Availability

No new data were created or analyzed in this study. Data sharing is not applicable to this article.

## References

[1] Varela P., Pio L., Brandigi E., Paraboschi I., Khen-Dunlop N., Hervieux E., Muller C., Mattioli G., Sarnacki S., Torre M. (2016). Tracheal and bronchial tumors. J. Thorac. Dis..

[2] Eyssartier E., Ang P., Bonnemaison E., Gibertini I., Diot P., Carpentier E., Chantepie A., Leclair M.D., Brouard J., Boutard P. (2014). Characteristics of endobronchial primitive tumors in children. Pediatr. Pulmonol..

[3] Gros L., Dei Tos A.P., Jones R.L., Digklia A. (2022). Inflammatory Myofibroblastic Tumour: State of the Art. Cancers.

[4] Iyer A., Radonic T., Heukamp L.C., Thunnissen E., Daniels J.M.A. (2021). Inflammatory myofibroblastic tumour of the central airways: Treatment and molecular analysis. ERJ Open Res..

[5] Dong Y., Zahid K.R., Han Y., Hu P., Zhang D. (2022). Treatment of Pediatric Inflammatory Myofibroblastic Tumor: The Experience from China Children’s Medical Center. Children.

[6] Coffin C.M., Hornick J.L., Fletcher C.D. (2007). Inflammatory myofibroblastic tumor: Comparison of clinicopathologic, histologic, and immunohistochemical features including ALK expression in atypical and aggressive cases. Am. J. Surg. Pathol..

[7] Schoffski P., Kubickova M., Wozniak A., Blay J.Y., Strauss S.J., Stacchiotti S., Switaj T., Bucklein V., Leahy M.G., Italiano A. (2021). Long-term efficacy update of crizotinib in patients with advanced, inoperable inflammatory myofibroblastic tumour from EORTC trial 90101 CREATE. Eur. J. Cancer.

[8] Kuo S.C., Lo Y.L., Chou C.L., Chung F.T., Lin S.M., Liu C.Y., Kuo H.P. (2015). Bronchoscopic debulking for endobronchial malignancy: Predictors of recanalization and recurrence. Thorac. Cancer.

